# Thermo-sensitive polymer nanospheres as a smart plugging agent for shale gas drilling operations

**DOI:** 10.1007/s12182-016-0140-3

**Published:** 2016-12-27

**Authors:** Wei-Ji Wang, Zheng-Song Qiu, Han-Yi Zhong, Wei-An Huang, Wen-Hao Dai

**Affiliations:** School of Petroleum Engineering, China University of Petroleum, Qingdao, 266580 Shandong China

**Keywords:** Nanoparticle plugging agent, Polymer microspheres, Thermo-sensitive polymer, Wellbore stability, Shale gas, Drilling fluid

## Abstract

Emulsifier-free poly(methyl methacrylate–styrene) [P(MMA–St)] nanospheres with an average particle size of 100 nm were synthesized in an isopropyl alcohol–water medium by a solvothermal method. Then, through radical graft copolymerization of thermo-sensitive monomer *N*-isopropylacrylamide (NIPAm) and hydrophilic monomer acrylic acid (AA) onto the surface of P(MMA–St) nanospheres at 80 °C, a series of thermo-sensitive polymer nanospheres, named SD-SEAL with different lower critical solution temperatures (LCST), were prepared by adjusting the mole ratio of NIPAm to AA. The products were characterized by Fourier transform infrared spectroscopy, transmission electron microscopy, thermogravimetric analysis, particle size distribution, and specific surface area analysis. The temperature-sensitive behavior was studied by light transmittance tests, while the sealing performance was investigated by pressure transmission tests with Lungmachi Formation shales. The experimental results showed that the synthesized nanoparticles are sensitive to temperature and had apparent LCST values which increased with an increase in hydrophilic monomer AA. When the temperature was higher than its LCST value, SD-SEAL played a dual role of physical plugging and chemical inhibition, slowed down pressure transmission, and reduced shale permeability remarkably. The plugged layer of shale was changed to being hydrophobic, which greatly improved the shale stability

## Introduction

At present, shale gas exploration and production has attracted much attention. Given its accumulation characteristics, extended-reach horizontal wells and cluster horizontal wells were drilled to produce shale gas. Because of the existence of micro-fissures and strong water sensitivity in shale formations, severe wellbore instability often occurs in the long horizontal sections, which seriously restricts the process of shale gas exploration and development (Cui et al. 2011; Dong et al. 2012; Wang et al. 2013). Shale formation is mainly composed of hard brittle shale, mainly of illite and mixed layer illite/smectite. For hard brittle shales, pore pressure transmission is the primary cause of wellbore instability. Therefore, the key to maintaining wellbore stability is to prevent pore pressure transmission. The effective sealing of micropores and micro-fissures is of great significance for preventing pore pressure transmission. Traditional plugging agents are difficult to form effective mud cake to prevent liquid penetration into shale matrix which has extremely low permeability and tiny pore throats. In recent years, nanoparticles are found to effectively plug shale pore throats to prevent liquid penetration into the formation, thus maintaining wellbore stability and protecting the reservoir (Roshan and Aghighi 2012; Rafieepour et al. 2013; Wen et al. 2014). According to previous experimental results, silica nanoparticles could significantly improve the densification of mud cakes, slow down pressure transmission and reduce shale permeability, while the rheology and lubrication of water-based drilling fluid were improved (Cai et al. 2012; Hoelscher et al. 2012; Al-Baghli et al. 2015). Bai and Pu (2010) synthesized PMMA latex nanoparticles with an average size of 73 nm, which can be used as a lubricant in drilling fluids based on their “ball bearing” function to prevent pipe sticking. They can also be used as a filtration reducer based on its deformability under temperature and pressure, forming a tough filter cake and sealing the micro-fissures in the formations drilled. Qu et al. (2007) synthesized intercalated or exfoliated nanocomposite poly(styrene-b-acrylamide)/bentonite using reversible addition-fragmented chain transfer (RAFT) polymerization. Experiments showed that these products had high-temperature tolerance and were good filtration control agents. In the last 20 years, the investigation into nanomaterials has greatly developed in many fields. Great progress has been made in basic theory and application of nanooptical materials, nanosemiconductor materials, nanobiomedical materials, nanoenhanced materials, nanomodified surface, etc. (Lin et al. 2012; Cormick and Hunter 2014; Kearnes et al. 2014). The combination of smart polymers with environmental response behavior (temperature, pH value, electrolyte concentration, magnetic field strength, electric field strength, etc.) and nanoparticles to realize the potential of nanoparticles is the most common research (Wu et al. 2013; Gulfam and Chung 2014; Lian et al. 2015). In this study, nanomaterials technology, smart polymers, and drilling fluid technology were combined. Emulsifier-free poly(methyl methacrylate–styrene) [P(MMA–St)] nanospheres with an average particle size of about 100 nm were synthesized in an isopropyl alcohol–water medium by the solvothermal method. Then, the thermo-sensitive smart polymer P(NIPAm–AA) was modified onto the surface of P(MMA–St) nanospheres and thermo-sensitive smart nanoparticles were obtained. With the change in temperature, the hydrophilicity and hydrophobicity of nanoparticle surface would change accordingly. Moreover, we would adjust the transformation temperature of NIPAm by an introduction of hydrophilic monomer or hydrophobic monomer, getting smart nanoparticles with different transformation temperatures to adapt to shale formations with different temperatures.

## Experimental

### Materials

Methyl methacrylate (MMA), styrene (St), and *N*-isopropylacrylamide (NIPAm) were purchased from the Aladdin Industrial Corporation and used after vacuum distillation. Acroleic acid (AA), potassium persulfate (KPS, K_2_S_2_O_8_), and tetrahydrofuran (THF) were purchased from the Sinopharm Chemical Reagent Co. Ltd and used without further purification.

### Preparation of thermo-sensitive poly(methyl methacrylate–styrene) nanoparticles

#### Preparation of poly(methyl methacrylate–styrene) latex nanoparticles

The latex particles prepared by emulsifier-free emulsion polymerization have good adhesion and good resistance to water. These latex particles are evenly distributed in a narrow size range with clear surfaces and relatively large particle sizes. Emulsifier-free emulsion polymerization was carried out by using a solvothermal method. A cosolvent-water mixture was used as the dispersed medium, and monomer polymerization was initiated in a closed system. The solvothermal method can improve the reaction temperature and pressure at the same time, so that the size of particles prepared in the medium decreased significantly, and the stability of the emulsion was improved (Hoa and Huyen 2013; Farooq et al. 2013; Mishra et al. 2014).

A total of 9.44 mmol MMA, 8.65 mmol St, and 0.3 mmol K_2_S_2_O_8_ were dissolved in a 38-mL isopropanol-water mixture, and the pH value adjusted to 7 by adding 1 mol/L NaOH solution. Then, the mixture was loaded into a PTFE lined hydrothermal synthesis reactor. After that, this mixed solution was stirred vigorously for 30 min and heated up to 90 °C. After heating for 1.5 h at 90 °C, the reaction mixture was diluted in 100 mL benzene. The mixture was precipitated and washed with methyl alcohol to remove the residual monomers and homopolymers. After drying for 8 h at 90 °C in a vacuum drying oven and grinding in a ball mill, poly(methyl methacrylate–styrene) [P(MMA-St)] nanoparticles were obtained.

#### Synthesis of thermo-sensitive polymer nanoparticles

**Fig. 1:**
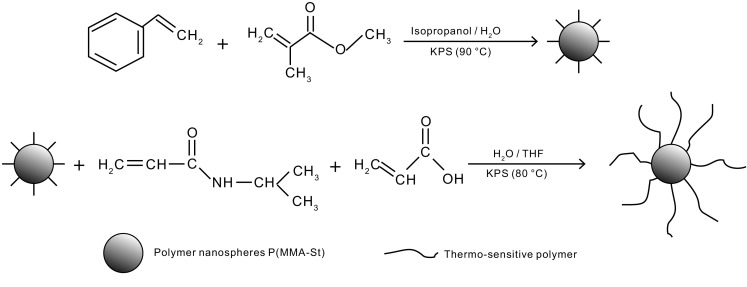
Synthetic route of SD-SEAL nanoparticles

A mixture of NIPAm and AA with a given mole ratio (no AA, 90/10, 80/20, 70/30, 74/26, 66/34, 52/48) was dissolved in an H_2_O/THF mixed solvent (the volume ratio of H_2_O and THF was 2:1), and then P(MMA–St) was added. The mixture was ultrasonically dispersed for 30 min. 0.2 mmol K_2_S_2_O_8_ was added dropwise, and the mixture was heated up to 80 °C and deoxygenated with N_2_ for 9 h. The synthetic route of the thermo-sensitive poly(methyl methacrylate–styrene) nanoparticles is shown in Fig. 1. The obtained product was centrifuged at 10,000 rpm for 30 min and washed with absolute ethyl alcohol to remove residual monomers. After centrifugation, the precipitates were collected, dried for 8 h at 90 °C in a vacuum drying oven and ground in a ball mill for characterization. The product was abbreviated as SD-SEAL.

### Structural characterization

The molecular structure of SD-SEAL was characterized by infrared spectroscopy which was recorded with a Nicolet 6700 FT-IR spectrometer (NEXUS, USA), scanning from 400 to 4000 cm^−1^ with a resolution of 4 cm^−1^ in transmission using KBr pellets. The KBr pellets were prepared by pressing mixtures of 1 mg of SD-SEAL powder and 100 mg of KBr. Transmission electron microscopy (TEM) measurements of SD-SEAL were acquired with a JEM-2100UHR electron microscope (JEOL, Japan). SD-SEAL solution with a concentration of 0.1 g/mL was dropped onto carbon-coated copper grids and dried in air. The microscopic morphology of shale was observed with an S-4800 field emission scanning electron microscope (Hitachi, Japan). The thermogravimetric analysis (TGA) of the SD-SEAL was performed on an SDT Q600 instrument (TA Instrument, USA). The sample was heated at a rate of 20 °C/min in nitrogen flow of 50 mL/min.

### Performance characterization

#### Temperature-sensitive behavior

There are a lot of methods to measure the temperature sensitivity of smart polymers. The most simple and commonly used method is to determine the light transmittances of a polymer solution at different temperatures (Feng et al. 2005; Kokufuta et al. 2012; Rwei and Nguyen 2014). When the temperature is lower than its lower critical solution temperature (LCST) value, the smart polymer is strongly hydrophilic. Its water solution is almost transparent, and the light transmittance is high. However, when the temperature is higher than its LCST value, the hydrophilicity of the smart polymer will be changed into hydrophobicity. At this time, micro-phase separation and turbidity will occur, so the light transmittance is almost zero. The curve of light transmittance as a function of temperature can be obtained after testing the light transmittances of the polymer solution at different temperatures. The temperature value corresponding to the inflection point of the curve is the LCST value of the polymer. That is the temperature corresponding to the light transmittance obviously declining. The temperature-sensitive behavior of polymer nanospheres (used as smart plugging agents) with different LCST values were acquired with an UV–Vis spectrophotometer (UV-1750, SHIMADZU International Trading Co., Ltd.).

#### Sealing performance evaluation

**Fig. 2:**
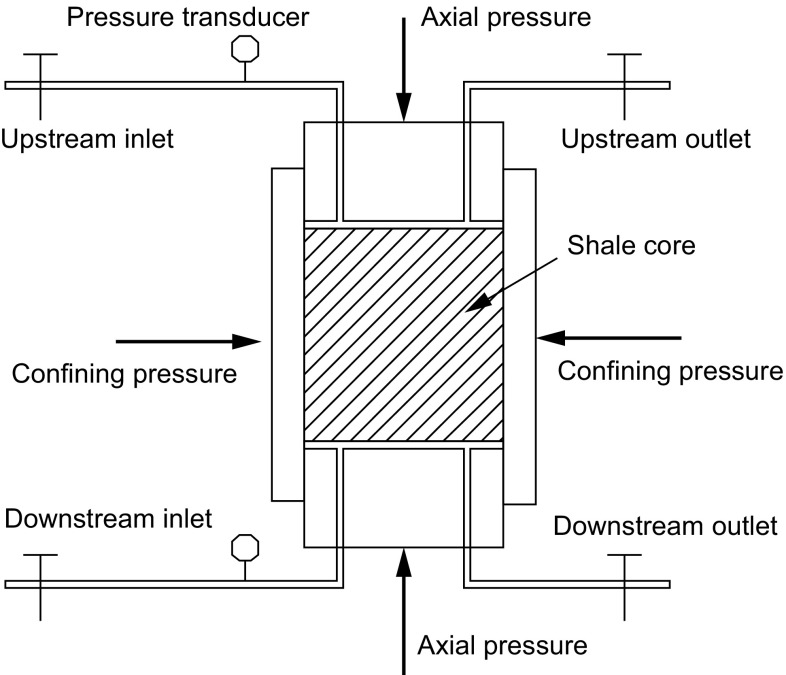
Schematic of pressure penetration test apparatus (Xu et al. 2005)

The pore pressure transmission test was used to measure the sealing performance of SD-SEAL using the simulation equipment for hydro-mechanics coupling of shale shown in Fig. 2 (van Oort 1994, 1997; Xu et al. 2005; Yuan et al. 2012). During pore pressure transmission tests, shale cores were installed in a core holder, and test fluids were pumped into the core holder from its upstream inlet to interact with the core. The confining pressure and the axial pressure were maintained at 5 MPa, the upstream pressure was maintained at 2.1 MPa, and the initial downstream pressure was 1.0 MPa. The pore pressure was determined by measuring the variation of the downstream pressure. Permeability of shale cores was calculated as follows (Xu et al. 2005):

1 $$K = \frac{\mu \beta VL}{A}\frac{{\ln \left( {\frac{{P_{\text{m}} - P_{\text{o}} }}{{P_{\text{m}} - P\left( {L,\;t_{2} } \right)}}} \right) - \ln \left( {\frac{{P_{\text{m}} - P_{\text{o}} }}{{P_{\text{m}} - P\left( {L,\;t_{1} } \right)}}} \right)}}{{t_{2} - t_{1} }}$$

where *K* is the permeability of the shale core, μm^2^; *μ* is the viscosity of fluids, mPa s; *β* is the static compression ratio of fluids, MPa^−1^; *V* is the enclosed volume of downstream fluids, cm^3^; *L* is the length of the shale core, cm; *A* is the cross-sectional area, cm^2^; *t* is the total experimental time, s; *P*_m_ is the upstream pressure, MPa; *P*_o_ is the pore pressure, MPa; and *P*(*L, t*) is the real-time downstream pressure, MPa.

#### Characterization of the core sealing surface

The microscopic morphology of the core sealing surface was observed with an S-4800 field emission scanning electron microscope (Hitachi, Japan). The wettability of the core sealing surface was measured with a JC2000D5M contact angle meter (Shanghai Zhongchen Digital Technic Apparatus Co., Ltd, China).

## Results and discussion

### Structural characterization of SD-SEAL

#### FT-IR

**Fig. 3:**
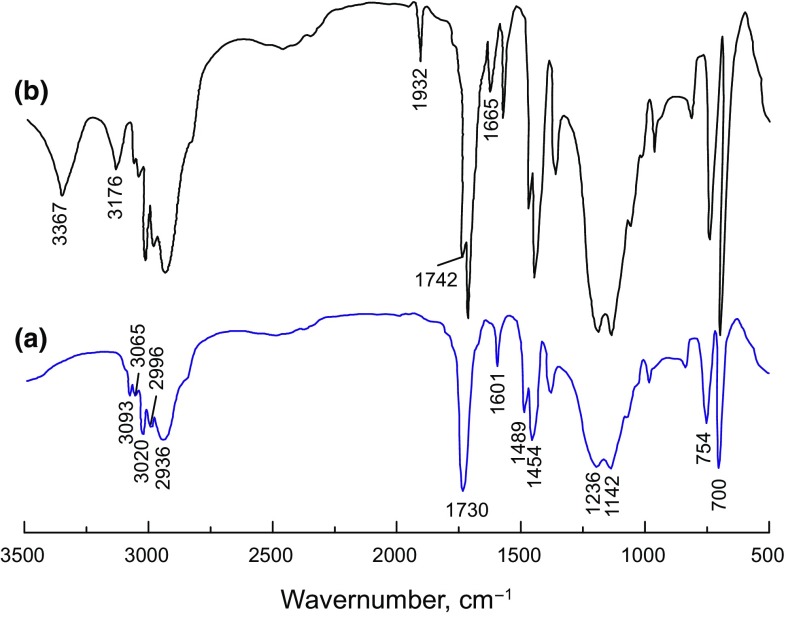
IR spectra of P(MMA–St) (**a**) and SD-SEAL (**b**)

FT-IR spectra of P(MMA–St) and SD-SEAL were shown in Fig. 3. For P(MMA–St), the absorption bands at 3093, 3065, 3020, and 2996 cm^−1^ were characteristic absorption peaks of C−H bond from monosubstituted benzene rings. The strongest absorption band at 1730 cm^−1^ was due to carbonyl stretching vibration. The absorption bands at 1236 and 1142 cm^−1^ could correspond to the symmetric stretching vibration of C–O–C bond. The absorption bands at 754 and 700 cm^−1^ were the characteristic bending vibrations of C−H from monosubstituted benzene rings. No absorption peak due to stretching vibration of C=C bond (1640 cm^−1^) was observed in Fig. 3a, meanwhile P(St) and P(MMA) homopolymers were separated before testing. Therefore, the above discussion confirmed that the newly synthesized latex particles were copolymers of St and MMA.

For SD-SEAL, except for characteristic peaks of P(MMA–St) copolymers, the absorption bands at 3367 and 3176 cm^−1^ were attributed to the stretching vibration of N–H bonds. The absorption bands at 1742 and 1655 cm^−1^ were characteristic absorption peaks of amide I (C−O bond) and amide II (N–H bond). An absorption band at 1932 cm^−1^ was the association absorption peak of −COOH. Since the synthesized products had been extracted with acetone, the homopolymers of AA and NIPAm were separated from the products, and the characteristic absorption peaks of NIPAm, AA and P(MMA–St) were obviously observed in the FT-IR spectra. So there were chemical bonds between P(MMA–St) particles and P(NIPAm–AA) polymers rather than a simple physical mixture. Namely, under certain reaction conditions copolymerization took place between P(MMA–St) latex particles and P(NIPAm–AA) polymers.

#### TEM

**Fig. 4:**
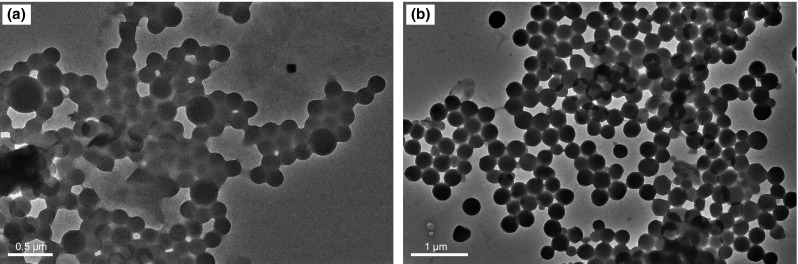
TEM images of P(MMA–St) (**a**) and SD-SEAL (**b**)

TEM tests on P(MMA–St) and SD-SEAL were conducted as follows: A small amount of P(MMA–St) or SD-SEAL was put into a dialysis bag for 24 h to remove the electrolyte ions in the products which allows only water molecules, ions and small molecules to pass through. The P(MMA–St) or SD-SEAL solution with a concentration of 0.1 g/mL was dropped onto carbon-coated copper grids and dried in air. TEM images (Fig. 4) of P(MMA–St) and SD-SEAL were acquired with a JEM-2100UHR electron microscope (JEOL, Japan). P(MMA–St) is a hydrophobic polymer, which is poorly dispersed in the aqueous solution, with irregular shapes and uneven particle sizes, and may form sticky agglomerates. SD-SEAL was well dispersed in the aqueous solution with regular shapes (mainly spherical) and uniform particle sizes (about 250 nm). Black spheres were observed in the center of the SD-DEAL particles, and the particle surfaces were covered with a thick gray polymer shell, indicating that thermo-sensitive polymer chains were successfully coated on the surfaces of P(MMA–St) nanospheres and products with core–shell structure were obtained.

#### Particle size distribution

**Fig. 5:**
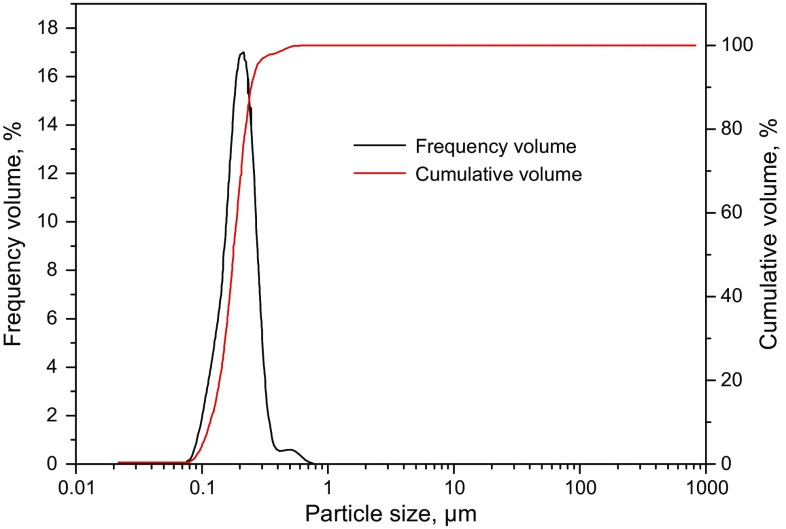
Particle size distribution of SD-SEAL

The particle size distribution and specific surface area of 0.001 wt% SD-SEAL solution were measured. As can be seen from Fig. 5, SD-SEAL had a narrow particle size distribution, mainly 90−360 nm, and had a *D*_50_ value of 252 nm, a *D*_10_ value of 179 nm, and a *D*_90_ value of 312 nm. Also SD-SEAL had a very large specific surface area, reaching 25,450 m^2^/kg, leading to a strong adsorption capacity. The particle size distribution of SD-SEAL was consistent with its TEM characterization results, further verifying that the synthesized products were successful. After flowing into pores and micro-cracks of shales, the coarse particles were prone to bridge and seal the larger openings of shales, and the finer particles were prone to fill the gaps between coarse particles. Finally, a dense sealing layer with low permeability was formed.

#### TGA

**Fig. 6:**
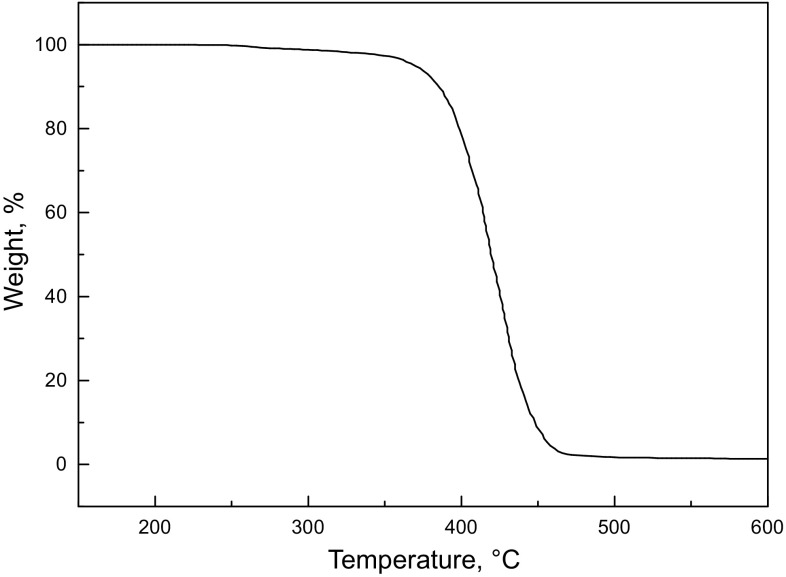
TG curve of SD-SEAL

The thermal decomposition of SD-SEAL was investigated by TGA (Fig. 6). The mass loss curve indicated two major stages. The first stage of mass loss occurred at around 200 °C corresponding to the evaporation of a small amount of adsorbed water and solvent (Mao et al. 2015; Zhong et al. 2015), while the second stage was the decomposition of SD-SEAL structures at around 380 °C, indicating that the newly synthesized products were highly temperature resistant, which was attributed to the presence of benzene in the SD-SEAL (Luo et al. 2016; Hu et al. 2016).

### Temperature-sensitive behavior

**Fig. 7:**
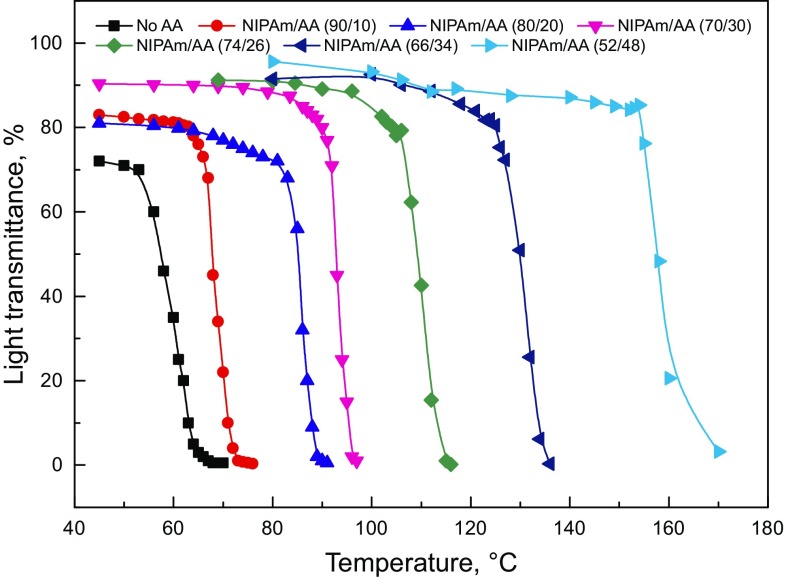
Transmittance of SD-SEAL as a function of temperature

The temperature-sensitive behavior of SD-SEAL was investigated by measuring the light transmittance of the SD-SEAL solution at different temperatures (Fig. 7). The experimental results showed that the light transmittance of the SD-SEAL solution dropped sharply when the temperature reached its LCST value, so SD-SEAL is temperature-sensitive. The LCST values of SD-SEAL increased with an increase in hydrophilic monomer AA. The LCST values were 53, 63, 81, 93, 106, 125, and 158 °C when the mole ratio of NIPAm to AA was no AA, 90/10, 80/20, 70/30, 74/26, 66/34, and 52/48.

The main driving force of the phase transition of SD-SEAL in aqueous solution was the hydrogen bond effect and hydrophobic effect (Huynh and Lee 2012; Chen et al. 2013; Xu et al. 2013). When temperature was lower than its LCST value, SD-SEAL had a high solubility in water due to its polar groups (−CONH− of NIPAm and −COOH of AA) on molecular chains. These polar groups interacted with surrounding water molecules to form strong hydrogen bonds. Because of the effect of hydrogen bonds and van der Waals force, the water molecules around the macromolecular chains would form solvation shells with high ordering degrees, which were connected by hydrogen bonds. So SD-SEAL can dissolve in water, and then its molecular chains can stretch in water, showing hydrophilic properties. When the temperature was above its LCST value, the hydrogen bonds formed between polar groups and water molecules were destroyed. Also the solvation shells of the hydrophobic parts of the molecular chain were destroyed, leading to an entropy increase in the dispersion system. The hydrophobic association of nonpolar isopropyl groups was dominant, showing hydrophobic properties of the whole molecule. Water molecules were expelled from solvation shells, causing phase separation. Therefore, with an increase in temperature, the regularity of hydrogen bonds was destroyed and the molecules were changed from hydrophilic to hydrophobic.

### Sealing performance

#### SEM observation of shale samples

**Fig. 8:**
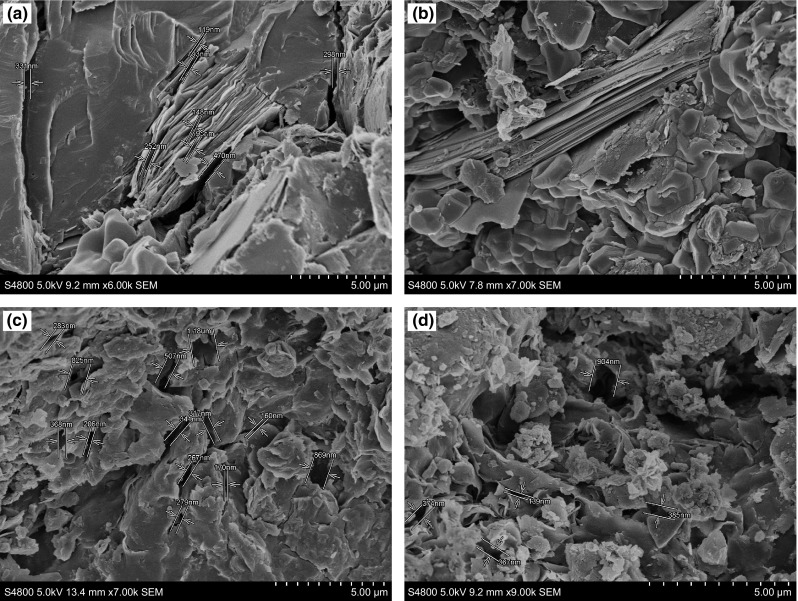
SEM images of shale samples from the Lungmachi Formation. **a** Micro-fissures. **b** Lamellae development. **c** Micro-fractures and micropores. **d** Micropores

The sealing performance of SD-SEAL was evaluated by the pressure transmission tests of shale samples collected from the Lungmachi Formation, Sichuan Basin. The microstructural characteristics of shale samples were observed by SEM (scanning electron microscopy). As shown in Fig. 8, the shale matrix is developed with parallel bedding planes, which are mainly formed by the dark organic layer and the organic-lean silicon layer. These bedding planes generally have a short horizontal extension and an intermittent development. Besides, their thicknesses range approximately from 200 to 500 μm. The SEM investigation results show that some micro-cracks and bedding planes are filled with the organic matter. The well-developed micro-cracks, which have widths about 0.5–3 μm, are significantly extended, bent, and partly show reticular distributions. The cracks are mainly distributed in the interior and edges of the rich organic matter layers, generally being parallel and vertical to these layers. Nanoscale pores (pore diameter, 200-800 nm) with poor connectivity are extensively observed in the shale matrix and organic matter.

#### Pore pressure transmission tests

**Fig. 9:**
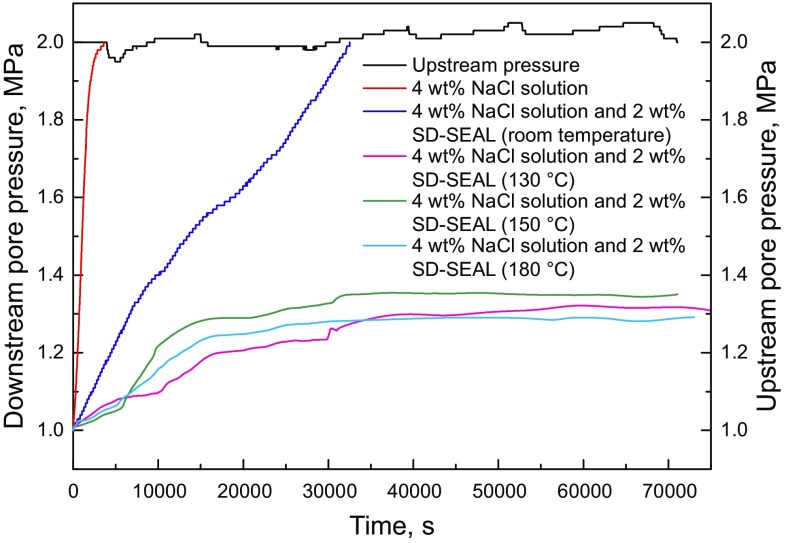
Pore pressure transmission tests of shale samples

In pore pressure transmission tests, the downstream fluid was 4 wt% sodium chloride (NaCl) solution, and the upstream fluids were 4 wt% NaCl solution, or a mixture of 4 wt% NaCl solution and 2 wt% SD-SEAL. The sealing performance of SD-SEAL (ratio of NIPAm to AA, 66/34; LCST, 125 °C) was tested at room temperature and temperatures above their LCST values (Fig. 9). The permeability of shale cores (Table 1) was calculated using Eq. (1).

**Table 1:** Permeability of shale cores

Test conditions	Shale permeability, 10^−7^ μm^2^
4 wt% NaCl solution	3.340
4 wt% NaCl solution and 2 wt% SD-SEAL (room temperature)	0.268
4 wt% NaCl solution and 2 wt% SD-SEAL (130 °C)	0.061
4 wt% NaCl solution and 2 wt% SD-SEAL (150 °C)	0.072
4 wt% NaCl solution and 2 wt% SD-SEAL (180 °C)	0.048

**Fig. 10:**
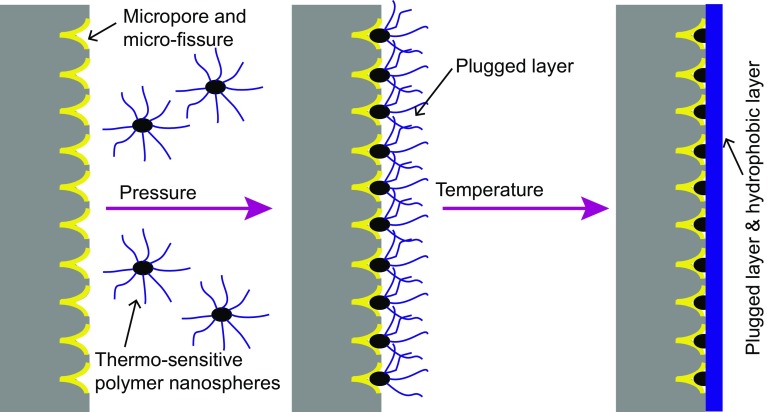
Schematic diagram of physical plugging and chemical inhibition of SD-SEAL

As can be seen in Fig. 9 and Table 1, the pressure transmission rate of brine increased very quickly and reached a steady state after testing for 6.5 min. At room temperature, SD-SEAL slowed down the pressure transmission rate and then reduced the shale permeability remarkably. Under the action of pressure, nanoparticles were pressed into micropores and micro-fractures in the shale surface, forming a physical sealing layer. The shale permeability was reduced from 3.34 × 10^−7^ μm^2^ to 0.268 × 10^−7^ μm^2^. When the temperature was above the LCST value of SD-SEAL, the downstream pressure change was small. 4 h later, the pressure transmission curve was close to a horizontal line. The effect of SD-SEAL slowing down pressure transmission and reducing shale permeability was much better. At this moment, the surface of SD-SEAL (thermo-sensitive polymers) changed from hydrophilic to hydrophobic. A hydrophobic layer was formed on the shale surface with an effect of water resistance. The shale permeability was reduced from 3.34 × 10^−7^ μm^2^ to less than 0.072 × 10^−7^ μm^2^, indicating that the SD-SEAL had a good temperature resistance. Therefore, SD-SEAL played a dual role of physical plugging and chemical inhibition when the temperature was higher than its LCST value, which greatly improved the shale stability (Fig. 10).

**Fig. 11:**
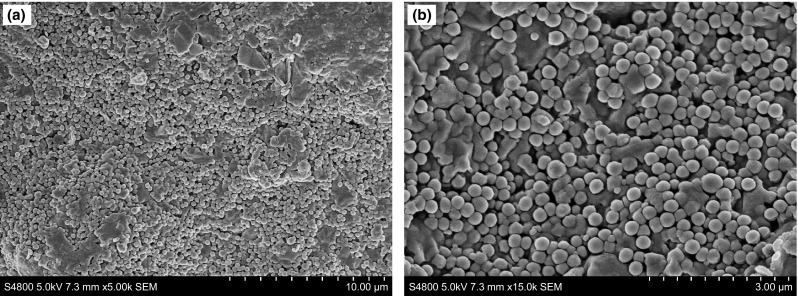
SEM images (**a**, **b**) of the surface of the plugged shale sample at different locations

The microstructural characteristics of the plugged layer of shale were observed with a scanning electron microscope. As can be seen in Fig. 11, after being sealed with SD-SEAL the shale surface was smooth and dense, SD-SEAL nanoparticles were tightly packed in micropores and micro-fractures in the shale sample, and spherical particles were clearly visible on the shale surface. This would significantly improve the core compaction and effectively reduce the core permeability.

### Wettability tests

The wettability of the plugged layer of shale was measured with a contact angle measurement. The wettability test results (Table 2) showed that the shale surface was strongly hydrophilic with a wetting angle of 12° before sealing. At room temperature, the plugged layer of shale was hydrophilic with a wetting angle of 38°. When the testing temperature was higher than the LCST value of SD-SEAL, the plugged layer of shale was changed to being hydrophobic. The wetting angle was 136°, 142°, and 139°, respectively, when the testing temperature was 130, 150, and 180 °C. The wettability test results further verified that SD-SEAL had a hydrophobic effect when the testing temperature was higher than its LCST value.

**Table 2:** Wettability test of the plugged layer of shale

Test condition	Wetting angle, °
Before sealing, room temperature	12
After sealing, room temperature	38
After sealing, *T* = 130 °C	136
After sealing, *T* = 150 °C	142
After sealing, *T* = 180 °C	139

## Conclusions

P(MMA–St) nanospheres with an average particle size of 100 nm were synthesized by the solvothermal method. Then, through radical graft copolymerization of thermo-sensitive monomer NIPAm and hydrophilic monomer AA onto the surface of P(MMA–St) nanospheres, a series of thermo-sensitive polymer nanospheres with different LCST values were prepared by adjusting the mole ratio of NIPAm to AA. The light transmittance of the SD-SEAL solution dropped sharply when the temperature reached its LCST value, so SD-SEAL is temperature-sensitive. The LCST values of SD-SEAL increased with an increase in hydrophilic monomer AA. The LCST values were 53, 63, 81, 93, 106, 125, and 158 °C when the mole ratio of NIPAm to AA was no AA, 90/10, 80/20, 70/30, 74/26, 66/34, and 52/48. When temperature was higher than its LCST value, SD-SEAL played a dual role of physical plugging and chemical inhibition, slowed down pressure transmission, and reduced shale permeability remarkably, which greatly improved the shale stability.
